# Surveying the citizen science landscape: an exploration of the design, delivery and impact of citizen science through the lens of the Open Air Laboratories (OPAL) programme

**DOI:** 10.1186/s12898-016-0066-z

**Published:** 2016-07-22

**Authors:** Linda Davies, Roger Fradera, Hauke Riesch, Poppy Lakeman-Fraser

**Affiliations:** 1 Centre for Environmental Policy, Imperial College London, South Kensington, London, SW7 1NA UK; 2 Department of Social Sciences, Media and Communications, Brunel University, London, Uxbridge UB8 3PH UK

## Abstract

**Background:**

This paper provides a short introduction to the topic of citizen science (CS) identifying the shift from the knowledge deficit model to more inclusive, participatory science. It acknowledges the benefits of new technology and the opportunities it brings for mass participation and data manipulation. It focuses on the increase in interest in CS in recent years and draws on experience gained from the Open Air Laboratories (OPAL) programme launched in England in 2007.

**Methods:**

The drivers and objectives for OPAL are presented together with background information on the partnership, methods and scales. The approaches used by researchers ranged from direct public participation in mass data collection through field surveys to research with minimal public engagement. The supporting services focused on education, particularly to support participants new to science, a media strategy and data services.

**Results:**

Examples from OPAL are used to illustrate the different approaches to the design and delivery of CS that have emerged over recent years and the breadth of opportunities for public participation the current landscape provides. Qualitative and quantitative data from OPAL are used as evidence of the impact of CS.

**Conclusion:**

While OPAL was conceived ahead of the more recent formalisation of approaches to the design, delivery and analysis of CS projects and their impact, it nevertheless provides a range of examples against which to assess the various benefits and challenges emerging in this fast developing field.

## Background

The term ‘citizen science’ is a broad term used to encapsulate a range of different activities, but in its essence, it partners professional scientists with volunteers in shared endeavour to study the physical and biological world. In this paper, we present an introduction to the historical context of citizen science (CS), and provide an overview of one programme cited as such, the Open Air Laboratories (OPAL) network, from concept through delivery and impact. We use OPAL as a framework against which to review the multifarious forms that citizen science activities may take. We compare current thinking on the design, delivery and impact of CS projects with experience gained from the OPAL programme and consider the contribution CS can make to broader scientific endeavour and societal concerns.

### Historical context of citizen science

#### The advent of ‘citizen science’

The contribution by members of the public to the collection, analysis and dissemination of scientific data is not a new occurrence. Volunteers, with no formal qualifications or affiliations, have contributed substantially to scientific discovery. The voluntary efforts of the ‘gentleman scientists’, such as Benjamin Franklin and Charles Darwin, made significant contributions to the advancement of scientific knowledge across a range of domains while making their living from other or private means [1].

Alongside individual enthusiasts, amateur societies, which have a long and rich history, have also provided mechanisms for public participation in science. Many societies provide forums to bring together professional and amateur members for fieldwork, education, promotion and conservation, while also actively encouraging and supporting involvement from the wider population. These opportunities have spanned a range of disciplines, with particular success in astronomy and environmental studies [2, 3].

While citizen-involved scientific activities continued throughout the twentieth century, there remained a division between the general public and those with high levels of expertise. That level of expertise could be acquired by citizens through the accredited training provided by the professionalised scientific realm or through the expenditure of considerable amounts of time, money and effort in self-directed study. Scientific expertise therefore remained the purview of a minority and those that gained it stood apart from the mass of society [4].

In this paradigm, the public generally had been conceived of as the passive beneficiary of scientific advancement and knowledge, without themselves having a particular voice in either the science itself or its policy applications because, being a lay audience, they lacked the necessary expertise to contribute. This “cognitive deficit” model is a term coined by Wynne [5] as a means of criticising this attitude towards the public (lack of) understanding of science and now is in widespread usage to refer somewhat disparagingly to old-style science communication. It diagnoses a deficiency in public knowledge and understanding of science and proscribes filling this deficit through processes where the public remains the recipient of scientific knowledge (with the process being one directional and educational in nature). Over time this view was challenged by studies that demonstrated the value of local and amateur knowledge to science [6–8] and the important contribution this can make to science policy. In parallel it was increasingly recognised that greater public science literacy does not automatically translate into more deferential support of expert opinion, nor a generally more enthusiastic public towards science [9].

#### The emergence of the term ‘citizen science’

The term “citizen science” was applied independently at about the same time in the United Kingdom and the United States (mid-1990s). Building on the developments outlined previously, citizen science was promoted by Irwin in the UK who, coming from a background of sociological research, envisioned a new strain of science where the professionals interact with the public to jointly formulate new knowledge and make informed decisions [9]. This tradition advocated a move away from the “deficit” model and instead emphasised that the public should engage with science rather than merely understand it, and also that scientists and experts need to be attentive towards the arguments and contributions the public can make towards science and scientifically informed policy. All this signalling that the communication between public and science should go both ways. As a result and alongside increasing recognition that society could and should play a more active role in the scientific process, new innovative science communication and other public engagement activities, such as science shops [10] and citizen juries [11], foreground democratic and active participation with experts developed. The aim was a critical two-way exchange rather than the mere transfer of knowledge from expert to public.

Independently of Irwin, however, the term “citizen science” was applied in the U.S. by Rick Bonney [12] to refer to a type of public engagement project that he and his colleagues were pioneering at the Cornell Laboratory of Ornithology. They aimed to combine the substantive tradition of amateur participation in ornithology research with an element of science communication and education targetted at those participating. This combination proved to be very successful and became an inspiration for the set-up of many similar projects both within the U.S. and abroad. Contemporary concepts of citizen science to an extent combine the aspirations of both, and citizen science activities arising from the tradition of Bonney can be seen as a possible way in which aspirations for Irwin’s citizen scientists can, in part, be realised.

#### Technological advancements supporting the growth of citizen science

Alongside changes in perceptions regarding the value to society of a more engaged, scientifically literate citizenship, technological advancements have transformed the public’s capability to contribute to scientific activities.

More powerful and internet-connected home computers have greatly increased the capacity of citizens to receive, collect and analyse data [1, 13]. The advent of the internet has improved communications, facilitated the development of new cultural processes, such as the crowdsourcing and sharing of data, and supported the growth of online networks of enthusiastic and interested participants [14]. The increasing sophistication of smartphones has turned every device into a potential mobile sensing station, with capabilities to record, interrogate and transmit global positioning system (GPS) location, time, images, acoustic information and other data [15–18]. Alongside increasing the capability of citizens to collect data, technology can also greatly improve confidence in those data. Sensors record data with known margins for error, while novel applications of existing technology can support data validation (for example, the submission of high resolution digital photographs for verification by experts) [19, 20].

While many new technologies supporting citizen science are ubiquitous in the developed world, technology can also promote participation in citizen science by citizens in less prosperous parts of the world. Sapelli [21], a mobile platform for data collection and sharing, was designed primarily for non-literate and illiterate users with little or no previous experience with computing technologies, supporting environmental monitoring by indigenous communities, which includes vulnerable groups with little involvement in the management of land on which they live [22].

### The OPAL programme

Open Air Laboratories (OPAL) was designed as an environmental education and research programme delivered through a national network of partners based originally in England (2007–2013) [23] and extended across the United Kingdom (2014-current).

#### Research and outreach drivers

The main scientific drivers behind OPAL were: (a) the objectives for sustainable development defined at the Rio Summit through the Conventions on Biological Diversity and Climate Change, and Agenda 21 [24]; (b) the UK crisis in taxonomy [25]; and (c) the decline in outdoor learning in the UK [26]. The unprecedented loss of global ecosystems [27] provided further evidence of the urgency of addressing these issues. Following the Rio Summit sustainable development was incorporated into the heart of UK government policy [28]. It was acknowledged then that government alone could not secure a more sustainable future and that everyone had a role to play. Community groups and the voluntary sector inter alia were identified as important participants in this endeavour. As sustainable development became more widely recognised so did the urgency of both the task ahead and the need for greater public awareness and engagement.

In the UK the National Lottery’s Big Lottery Fund [29] is recognised as a leading supporter of programmes that improve social well-being and address relevant policy areas. In 2005 they established a major new funding initiative, Changing Spaces, calling for environmental projects that would educate and engage local communities in sustainable development. Emphasis was placed on supporting disadvantaged communities in their local environment but the programme was designed to reach all sectors of society. OPAL was therefore conceived in response to a recognised policy need (sustainable development and the environment) and funded by a national public body.

#### Concept

In response to this call in 2005, Imperial College London (ICL) proposed a very simple concept: take scientists out of their institutions and into the heart of the community to share knowledge and engage local communities in field-based research.

The three research topics relevant to the identified research and outreach drivers were: pollution (air, water and soil), loss of biodiversity and climate.

The majority of OPAL-England partners were research scientists who were used to meeting regularly to share knowledge and develop collaborative research. They were joined by representatives from local and national government and their agencies and leading environmental organisations, such as the Natural History Museum (NHM), as well as community organisations affected by environmental issues such as the impact of air pollution and loss of biodiversity (parks and conservation managers). These meetings were initially funded through a network grant provided to ICL by the Engineering and Research Council [30] for the Air Pollution Research in London (APRIL) network in 1999 [31]. Davies established the APRIL Natural Environment Group from which the OPAL proposal emerged (APRIL is now managed by the Greater London Authority). The OPAL partnership was therefore largely already established as a collaborative research network familiar with research and policy needs (drivers).

Reflecting the aims and objectives of the Big Lottery Fund, the programme sought to engage a wide audience, particularly people from disadvantaged sectors of society, people not previously engaged with nature, as well as the general public. All partners recognised and supported these aims although for many it was their first experience of working directly with the public.

#### Funding

ICL was initially awarded £11.8m by Big Lottery Fund to direct and manage the OPAL programme, with additional funds (£1.3m 2010; £1.4m 2012) awarded in subsequent years as the impact of the public participation activities was recognised. In 2014 further funding (£3.0m) was awarded to extend the community engagement work across the UK (OPAL-UK). An overall goal was agreed initially of one million beneficiaries comprising 500,000 through field studies and 500,000 online (with a further 100,000 in-field beneficiaries to be delivered through the OPAL-UK programme). Other targets were agreed and a range of quantitative data was gathered throughout the programme, for example demographic data (i.e. percentage of disadvantaged communities reached and age ranges of participants), media circulation data, web visitors etc., whilst qualitative data were gathered through comment boxes on the website, online and in-field questionnaires, and by social scientists employed to work on the programme.

#### Goals

There were five key objectives:

1. Supporting a change of lifestyle, a purpose to spend time outdoors, observing and recording the local environment;
2. Developing an exciting and innovative educational programme that can be accessed and enjoyed by all ages and abilities;
3. Inspiring a new generation of environmentalists;
4. Gaining a much greater understanding of the state of the natural environment for research and policy purposes;
5. Building stronger partnerships between the community, voluntary and statutory sectors.

#### Programme design and structure

**Fig. 1:**
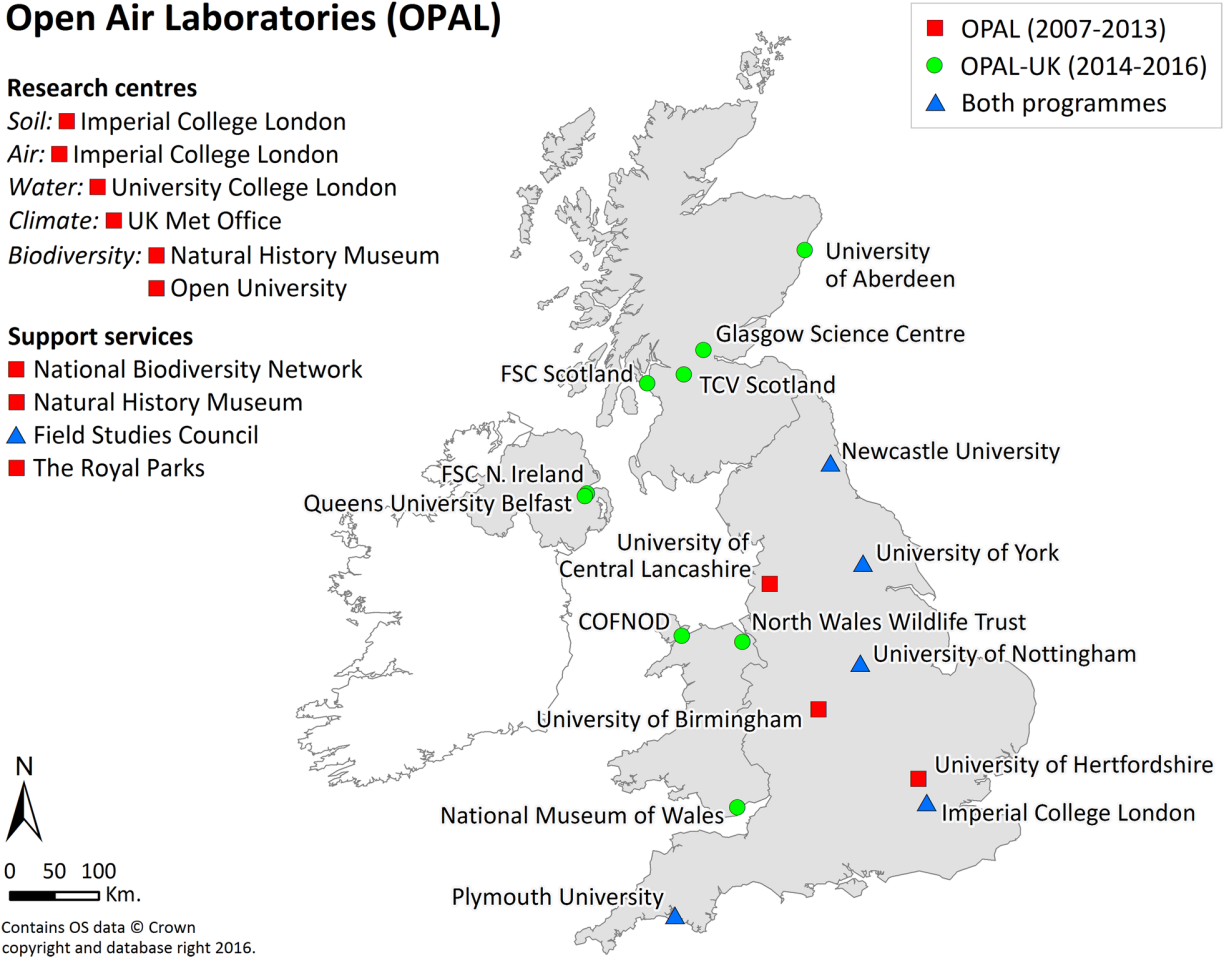
Funded partners in the Open Air Laboratories (OPAL) network. Geographic locations of regional partners with engagement staff (Community Scientists) are displayed on the map and partners leading national research centres and providing essential supporting services are listed to the *left*. The period during which partners were active in the OPAL network is indicated

The OPAL network is illustrated in Fig. 1. ICL directed and managed the programme guided by an external Advisory Board and supported by a series of regional and (under OPAL-UK) national committees that sought to coordinate activities and, in doing so, maximise programme impacts and support the OPAL objective to promote stronger partnerships between the community, voluntary and statutory sectors.

Under the original programme, OPAL established nine regional teams. Each was based in a university and worked directly with local people on research and educational projects of relevance to their region. Community Scientists, a new role created for the programme, worked under the direction of the regional lead scientist and, together with the schools programme [led by the Field Studies Council (FSC)] and public parks programme (led by the Royal Parks), were the main public engagement mechanisms, motivating and engaging local people. Under OPAL-UK, new partners extended public engagement activities to Scotland, Wales and Northern Ireland.

OPAL initially set up research and educational centres (Fig. 1) to provide scientific expertise, carry out research with varying degrees of public engagement (science workshops, public demonstrations, training days, publications in plain English, online progress reports, blogs and attending local and national fairs and events), and deliver research and educational tools. They also led the design and analysis of the OPAL field surveys, OPAL’s primary citizen science activities. A large support service underpinned the programme including a national media strategy, web services, data management, and publications.

#### Engaging participants outdoors

It was recognised that deprived communities and people from disadvantaged sectors of society were less likely to engage through mainstream media or traditional approaches to public engagement in science so a significant proportion of staff time was spent working to engage these groups. The Index of Multiple Deprivation [32] helped to identify areas to target work and guidance from local authorities and local voluntary sector representatives, including those represented on OPAL regional and national committees, also helped Community Scientists to make contact with minority groups. These and many other innovative approaches were used to build relationships of trust with local communities through repeated face-to-face contact.

#### Engaging participants through digital tools and media

In addition to the significant staff resources (the original programme comprised fifteen organisations and over 100 staff employed in either full, part-time or in voluntary capacities) used to achieve OPAL’s direct participation objectives, digital tools and traditional media services were used to reach the general public.

The OPAL website [33] provides the main interface for all participants. It houses the OPAL database where all public data are initially submitted, provides instant feedback through interactive visualisations and mapping, as well as presenting research findings in plain English. It also contains all of the educational materials OPAL has developed (free to download), blog posts on community achievements, scientist profiles, and topical news. Further digital projects, such as iSpot and Indicia, were also developed as part of the programme (see below).

A media strategy was designed and led by OPAL partner, the NHM, with their extensive experience of public engagement and all partners, staff and students were encouraged, trained and supported to contribute.

### Classifying citizen science

Citizen science has grown to the extent whereby an understanding of the breadth of projects classified as CS can be helpful to drive the field forward. While elements of volunteer involvement in science have been practiced for centuries, Silvertown [1] notes that the modern use of the term citizen science has only been recognised relatively recently. For example, in January 2009 only 56 articles in the search engine ISI Web of Knowledge were explicitly tagged with the term ‘citizen science’; by January 2016 this had risen to over 11,000. Academic publications are not the only indication of the rise of citizen science; the discipline has now reached a maturity where there have been various conferences [34, 35], interest groups [36] and, membership organisations [37–39], seeking to share best practice among practitioners. As the concept of CS has developed a number of classification models have been proposed to understand the diversity of the practice.

At the broadest conceptual scale, Dickinson and Bonney [40] proposed four axes along which environmental CS varies: initiator of projects (academics or public), scale and duration (global/local, short term/long term); types of questions (pattern detection to hypothesis led); and goals (research, education and stewardship).

Reflecting a number of these axes, Prainsack [41], working mainly from the perspective of medical citizen science projects, distinguished them along a number of different dimensions. These include, for example, who has the ability to set the agenda, how the project affects local communities and how open it is with the resulting data and scientific research. Haklay et al. [42] propose a classification framework based on the level of participation from citizens: those requiring the least involvement as (i) ‘Crowdsourcing’, whereby citizens volunteer computing power or provide and maintain sensors; next (ii) ‘Distributed intelligence’, whereby the cognitive abilities of participants is utilised to collect or interpret basic data, sometimes with more limited, prior training; next (iii) ‘Participatory sensing’, where citizens are involved in problem definition and work with scientists to design a data collection methodology; and finally (iv) ‘Extreme citizen science’, where the relationship between scientist and citizen is collaborative, with opportunities for citizen involvement at all stages of the scientific process, with professional scientists acting “as facilitators, in addition to their role as experts” (p. 12). Wiggins and Crowston [43] identified five types of citizen science projects, including action projects (instigated by the local community to address matters of civic concern), virtual projects (based on internet contributions), investigation projects (driven by scientific aims requiring data collection from the physical world), conservation projects (promoting stewardship of natural resources), and education projects (focusing on education and outreach through formal and informal learning opportunities). For example, some celebrated internet-based and science led projects such as Galaxy Zoo [44] would in this classification fall under both virtual and investigation type.

OPAL, conceived in 2005, can be considered a pioneer in the application of large-scale CS even though it was not explicitly designed to any established framework of criteria for CS. We utilise the aforementioned broad conceptual framework of Dickinson and Bonney [40] (which encapsulates many other more detailed classification systems) and draw on examples from OPAL to investigate the breadth of citizen science in this section.

#### Initiator of project

Along one of Dickinson and Bonney’s four axes—initiator—the Centre for the Advancement of Informal Science Education (CAISE) [45] propose three categories for citizen science projects based on the amount of control that participants have over the different steps of the activity: (i) Contributory projects, where the activity has been designed by professional scientists and to which citizens are invited to contribute data as per the specified methodology; (ii) Collaborative projects, where scientists still lead the project but citizens are invited to refine the design of activities, analyse data, or disseminate findings; and (iii) Co-created projects, where the activities are designed by scientists and citizens working together and “public participants are actively involved in most or all steps of the scientific process” [45].

OPAL is policy driven and the majority of research questions were formulated by academics, their students, or collaborating organisations, and therefore citizens, in the main, acted in a contributory fashion, providing data they collected to answer research questions and using methodologies as defined and developed by professional scientists. OPAL’s main mechanism for engaging the public occurs when public participation is intrinsic to research methodology (although not the research questions), namely the national field survey series (the OPAL surveys). The OPAL surveys allow people to work independently at a time, place and pace of their choosing, or directly in the field with OPAL Community Scientists (or other groups trained by OPAL) providing guidance and support. OPAL has developed seven surveys to date (and several mini surveys), each focusing on a different environmental topic (Table 1). The surveys often use biomonitoring within their methodologies, an approach long used [46] whereby selected biological organisms can provide information on the state of their environment. OPAL surveys include equipment such as strips for pH measurements and tape measures as well as laminated, illustrated, instruction cards (with policy links and health and safety advice). In terms of their intended audience, the surveys were designed with an educated 13–14 year old in mind or adults new to environmental issues, however younger or less able participants can take part with appropriate support or with materials suitably adapted. Survey data are entered directly by participants to the OPAL database via the OPAL website and analysed by the lead scientist for that topic. When the first OPAL survey was launched (OPAL Soil and Earthworm Survey, 2009) lack of access to a computer proved a problem so a free post address was introduced.

**Table 1:** The OPAL national citizen science surveys

Survey name	Launch date	Aim	Approach	Output examples
OPAL Soil and Earthworm Survey	2009	Which species of earthworm are found in which soil and habitat types	1. Assessment of site characteristics 2. Assessment of soil properties 3. Earthworm ID	Hypothesis led and policy links e.g. [71]
OPAL Air Survey	2009	Bio-indicators assessing local pollution and distribution of lichens and Tar spot on Sycamore	1. Assessment of site characteristics 2. Assessment of tree characteristics 3. Identification of indicator lichens/fungus	Hypothesis led e.g. [56]
OPAL Water Survey	2010	Water quality of ponds	1. Assessment of site characteristics 2. Assessment of water clarity 3. pH test 4. Identification of indicator invertebrates	Hypothesis led e.g. [59]
OPAL Biodiversity Survey	2010	Condition of hedges	1. Assessment of site and hedge characteristics 2. Assessment of food resources 3. Identification of invertebrates 4. Tracking presence of other species	Hypothesis led: e.g. [70]
OPAL Climate Survey	2011	Human activities and climate	1. Observations of aircraft contrails 2. Measurement of wind speed and direction 3. Thermal comfort	Validation e.g. [57]
OPAL Bugs Count Survey	2011	Impact of a changing environment on urban and rural areas	1. Assessment of site characteristics 2. Assessment of microhabitats 3. Identification of invertebrates	Distribution monitoring e.g. [60]
OPAL Tree Health Survey	2013	Condition of trees and the pests and diseases that affect them	1. Assessment of site characteristics 2. ID of common pests and diseases 3. ID of threatening pests and diseases	Policy requirement: e.g. Defra strategy [58]

The OPAL surveys were designed to provide a low technology approach to citizen science (and thus reducing barriers to participation, particularly for groups from lower socioeconomic backgrounds, a focus for OPAL’s engagement); the opportunity to exploit new technologies and develop digital communities was, however, recognised as an important mechanism for OPAL to deliver its objectives. Some activities were undertaken in response to social and technological developments; for example, the arrival and increased public ownership of smartphones led to developing OPAL survey data submission via mobile phones (first used in 2011 for the OPAL Climate Survey) and a first app (in 2012 for the OPAL Bugs Count Survey). However, an integrated series of digital projects that sought to exploit crowdsourcing capabilities while building a new digital community was built into the OPAL programme by design.

iSpot [47] an online, interactive social network aimed to help the public to correctly identify wildlife and to build and reward the development of taxonomic skills. Participants share photographs of wildlife on the website and a community of amateur experts and professional scientists then provide participants with either verification of their identification or propose new identifications. The online experts providing support were initially OPAL-funded staff members but natural history societies very quickly became interested in the data being submitted by the public and, increasingly, as non-expert users developed their taxonomic skills, they also contributed to verification of records submitted by other users; in so doing iSpot could be considered a CS project that can span both of the CAISE classifications of contributory and collaborative CS. iSpot to January 2016 had >55,000 registered users who supported the identification of >700,000 records (personal communication, Janice Ansine). More than half of the submissions were identified within an hour (and >80 % were named to species level) [48].

While the majority of OPAL’s CS activities would fall into contributory or, perhaps, collaborative classifications, there are examples where co-created or entirely citizen-led CS has occurred, often developing organically from OPAL activities. For example, staff members at the OPAL Yorkshire and Humber regional project (University of York), together with a local ranger, were interested in working with local people to monitor the colonisation of flora and fauna onto an ex-coalfield site in Wakefield. This work identified that the pond on the site was infested with invasive crayfish. The local Anglers Association who managed the site were keen to find a way to manage the invasive species and contribute to furthering understanding of this species (as well as others on the site) and so with OPAL staff they applied for a scientific trapping license from the Environment Agency. Another example is La Sainte Union Catholic School, which first used the OPAL Air Survey packs to study local air quality and lichens. The school then contacted the British Lichen Society (BLS) through OPAL and worked with them to develop a project that was awarded a partnership grant by the Royal Society to investigate the relationship between air quality and lichen distribution. Using diffusion tubes they measured levels of nitrogen dioxide as a means of validating the OPAL pollution index based on lichen indicator species employed by the OPAL Air Survey [49].

#### Project scale and duration

Revisiting Dickinson and Bonney [40] we look now at spatial and temporal scales and how they can vary between citizen science projects. Some projects may last just one field season (e.g. the Big Bumblebee Discovery [50]) whereas others have continued for decades (e.g. the Christmas Bird Count [51]); some may encourage citizens to examine their local area (e.g. the Hackney Wick Community Map [52] which allowed communities around Hackney Marsh, London to map a site less than 2 km^2^) while others provide platforms for citizen scientists to work across continents (e.g. iNaturalist [53]).

As part of the OPAL-UK programme, and following testing with local communities for cultural variation and the relevance of indicator species, the seven OPAL surveys were adapted and extended across the UK (including translation of materials into Welsh language) in 2015. However, before this funding was awarded data had already been received from these countries (nearly 800 sites had previously been surveyed). Furthermore OPAL survey data have been received from many other European countries and further afield. Not all survey methodology is transposable to these areas although some data may remain valid (e.g. physical or chemical conditions) and regardless of the research value, participants may receive educational and stewardship benefits. After almost 10 years in operation public participation in OPAL remains high. Efforts to sustain OPAL core activities are ongoing and remain challenging.

#### Types of research

Just as the research objectives underpinning citizen science activities can vary from hypothesis-led investigations (Conker Tree Science [54]) to pattern recognition exercises (Galaxy Zoo [55]); the approaches and types of scientific questions underpinning each of the OPAL surveys varied considerably.

Table 1 summarises the main type of research questions posed by the OPAL national surveys. These span the range of question types identified by Dickinson and Bonney [40]. For example, the OPAL Air Survey involved elements of hypothesis-led work, investigating whether fungi could be used as a bio-indicator of air pollution [56]. The study partly seeks to understand whether Tar spot fungus appears less frequently on sycamore trees in urban areas than in rural areas where air pollution levels and leaf litter management practices differ. Other studies used publicly collected data for validating computer model predictions: for instance, in the OPAL Climate Survey participants submitted observations of aircraft contrails which were then compared against model predictions of humidity levels at aircraft height [57]. The OPAL Bugs Count Survey placed more emphasis on monitoring species distribution change in urban and rural environments. In addition to scientific questions, environmental policy drivers directly shaped the design of surveys: the OPAL Soil and Earthworm Survey was developed in part to examine whether citizens could contribute data to support soil condition assessments and the OPAL Tree Health Survey supported official government monitoring of tree pests and diseases [58].

To ensure that the quality of data collected was of a usable standard, each OPAL survey was developed through a working group chaired by a scientific lead, supported by other scientists, representatives from natural history societies, government agencies, and other stakeholders. The process involved experts in graphic design, education, communication, web design, social science and public engagement. Drafts were regularly circulated to all OPAL staff for comments and tested with the community before final publication. Mechanisms to minimise error and to help validate records were introduced throughout the programme and ranged from collecting photographic evidence of observations from participants to online quizzes to determine the level of skill of the participant and weighting of data at the analysis stage [59, 60].

The development and delivery of citizen science does not occur in isolation from the social, political and economic conditions surrounding its goals of outreach and research. OPAL contained discrete supporting projects that did not necessarily constitute citizen science of themselves, but were considered to be essential to the processes of enabling citizen science. In response to the acknowledged ‘crisis’ caused by the then shortage of skilled taxonomists [25], OPAL sought to raise awareness and to increase the profile of natural history societies and conservation groups (voluntary sector) who play a critical role in biological recording and education. The OPAL programme included a dedicated funding scheme, led by the NHM, to help these groups to modernise, recruit new members and raise the profile of their societies with the public. Seventy organisations were awarded grants and a new web interface and database [61] was designed detailing their expertise and their contact details. Many of these organisations provided support to OPAL, particularly to the Community Scientists. At that time no natural history society existed dedicated to the study of earthworms, the biological element within the OPAL Soil and Earthworm Survey, so the Earthworm Society of Great Britain was established in 2009 through an OPAL grant. The society has the aims of (i) conducting research into earthworms; (ii) promoting knowledge and appreciation of earthworms within the non-scientific community; and (iii) educating the non-scientific community in earthworm biology and ecology. The organisation has now established the National Earthworm Recording Scheme and is in the process of developing distribution maps for the 27 species of earthworm; it has also run public events, provided identification training courses and has also developed its own citizen science survey (the Earthworm Compost Survey [62]), thereby continuing to support and foster the conditions for public participation in earthworm ecology research (personal communication, Kerry Calloway).

New software was developed to encourage and facilitate biological recording. The National Biodiversity Network (NBN) [63] manages the national database for biological records in the UK. Through OPAL funding, free, open-source biological recording software known as Indicia was developed [64] and is used by more than 80 societies in the UK and abroad. Indicia required a level of skill beyond that of most OPAL participants and so an easier to use version of Indicia known as Instant Indicia was developed, and also an implementation of Indicia known as iRecord [65], which was designed to allow any member of the public to create their own biological recording account and begin submitting observations of nature. Uptake of iRecord has been extremely positive, with the millionth record submitted in September 2015 [66].

#### Setting and achieving goals

Defining objectives and monitoring progress against them are important components of any CS programme and are the final classification of Dickinson and Bonney [40]. The OPAL Community Environment Report [67] prepared for the funding body and participants alike summarises OPAL’s achievements over the first 4 years of operation covering preliminary research findings, unexpected outcomes, lessons learned and tools and materials designed. All targets agreed with the funder at the outset of the original programme were achieved or exceeded (separate but related outcomes were agreed for the OPAL-UK programme to be delivered by the end of the programme in December 2016). Table 2 provides a summary of the programme’s delivery against its targets, updated since publication of the Community Environment Report, with data following completion of the first 6 year programme.

**Table 2:** OPAL original programme (2007-2013) impact data

Objectives	Target impact	Delivered impact	Source
1. Spending time outdoors, observing and recording	a. Engagement with 500,000 participants at field events b. Engagement with 500,000 online visitors	a. >850,000 participants at field events (>20 % of regional project engagement with disadvantaged beneficiaries) b. >540,000 visitors to the OPAL website; >1.1 m visitors to iSpot; >520,000 visitors to Indicia sites or iRecord	a. Data provided monthly by OPAL staff b. Web generated data (NHM/ICL; OU; NBN)
2. Creating an educational programme	240,000 survey packs to be designed, printed and distributed	>275,000 survey packs designed printed and distributed; c50,000 educational resources downloaded	Print run data (FSC) and distribution data (OPAL partners); Web generated data (NHM/ICL)
3. Inspiring a new generation of environmentalists	a. Increasing access to natural history societies (membership at 10 societies increased by 10 %) b. working with schools	a. 32 (46 %) of societies monitored >10 % increase in membership b. working with 3100 schools	a. Data collected by NHM through monitoring associated with OPAL grants programme b. Data provided monthly by OPAL staff
4. Supporting a greater understanding of the state of the environment	No numerical target	>30,000 field surveys submitted (>22,000 further observations of contrail observation sub-activity) >20 research manuscripts citing OPAL methods, using OPAL data or supported by OPAL funding	OPAL website survey entries Publications
5. Building stronger partnerships between voluntary, community and statutory sectors	a. Raising awareness through media engagement > 500,000 b. Engaging with community groups (no numerical target)	a. National coverage by >180 radio, TV and print media hits; >100 websites; total circulation figures exceeded 100 million b. Working with >2400 organisations	a. Media cutting service managed by OPAL Communications Project (NHM) b. Data provided monthly by OPAL staff

Below, we summarise further examples of impact across the goals for research and outreach. Looking at the interface between these goals, Lakeman-Fraser et al. [68] assimilate the quantitative and qualitative evaluation throughout the original 5 year programme drawing out trade-offs associated with multiple aim projects and identifying key considerations to tackle these challenges when planning and delivering a citizen science project.

##### Research

Research outputs span environmental and social science fields [69]. Taking an ecological approach Rose et al. [59], for example, analysed the data collected from the OPAL Water Survey yielding a national assessment of water quality and clarity in England, whereas Gosling et al. [70] investigated the OPAL Biodiversity Survey finding that urban hedges as well as rural hedges can be important habitats for wildlife. A host of other manuscripts have been produced on the scientific outputs of the OPAL programme [56, 59, 60, 71] and the methodological considerations when monitoring data quality [57, 60, 72].

Exploring the societal impacts of citizen science Everett and Geoghegan [73], for example, investigated the motivations and barriers that people face initially engaging with a programme and maintaining enthusiasm for that programme. Other research into this area focused on people from socio-economically deprived backgrounds getting involved for the first time [74], issues that scientists are faced with when getting involved in citizen science [75, 76] and the education and behavioural impact of citizen science involvement [67].

Through OPAL more people have now engaged in activities related to environmental policy and sustainable development objectives (OPAL drivers), particularly with regard to Agenda 21 and the Convention on Biological Diversity (Articles 7,12,13) which promote monitoring, research, education and public awareness [77]. By working together scientists and the public have gathered a wealth of new data about wildlife, its distribution across England and the condition of their habitats. Some of these data are from sites that have previously been difficult for scientists to access such as gardens and inner city areas, allotments and playing fields.

##### Outreach

The OPAL mantra is ‘Explore Nature’ and is all about encouraging people to get outside and learn about the environment on their doorstep. OPAL sought to engage all parts of society through a range of different approaches. For example, media coverage spanned national and local newspapers, television, radio, and online news sources: for example, the OPAL Soil and Earthworm Survey was reported on by, amongst others, the BBC One Show (estimated viewing figures of 4.8 million), BBC Radio 4 (estimated listening figures of 3.3 million), and the Daily Mail (estimated circulation figures of 2.2 million) [78].

Reflecting its funder’s mission to focus on “communities and people most in need” [29], particular effort was placed on engaging communities without a large tradition of participation in scientific research, such as those from deprived, low-social capital areas. Traditionally, such groups tend to have fewer cultural resources to fully participate in local environmental decision making, or the social capital to make their voices heard above those of the experts, compounded by a lower access to high level education. Evidence also suggests that groups from lower socio-economic backgrounds tend to live in areas of lower environmental quality (e.g. [79]) and therefore may have greater need to participate in environmental decision making. Citizen science therefore presents a powerful mechanism through which to raise awareness and engage local people in local issues.

Social data taken from the OPAL Community Environment Report [67] indicate that: Half of all participants submitting survey data to the website (8450 from a sample of 16,766 people) state that this was the first time they had carried out a survey; just eight percent (695 from a sample of 9261) said they would not carry out another survey; almost half (43 %) of people interviewed (254 from a sample of 593) said taking part had changed the way they thought about the environment; more than a third of this groups (37 %) said they would change their behaviour towards it; 90 % of participants (13,142 from a sample of 14,621) said they had learnt something new; 83 % of these respondents said they had developed new skills. Approximately 20 % of engagement delivered by OPAL Community Scientists has been with individuals who classify as deprived or in some way hard-to-reach.

A wide range of materials have been developed across all topics for all ages and abilities and are stored on the OPAL website. They are widely used by schools, universities and other educational organisations such as the British Science Association. OPAL has worked with >3100 educational establishments (54 % secondary schools; 43 % primary schools; 2 % universities; and 1 % special schools) and school children contributed survey data relating to c15,000 sites (50 % total submissions). 10 % of the primary schools involved were located in the most deprived 10 % of England (6 % of survey results came from these areas). iSpot continues to be widely used and has been incorporated within the Open University’s OpenScience Laboratory initiative that seeks to make practical science available to any student with a connection to the internet [80].

Contributing to a national research programme was a key motivating factor for many participants. OPAL’s high quality science programme was said to give confidence to both teachers and students to carry out more fieldwork. Unplanned positive impacts on health and well-being were reported by many group leaders and participants during the programme. The high level of interest from schools was another unexpected outcome with many citing the outdoor learning programme and the opportunity for pupils to contribute to real research and to work with scientists as important factors.

## Conclusions

The OPAL programme provides an encompassing case study that spans a range of approaches within the citizen science landscape. OPAL can be viewed retrospectively as contributing to the democratic ideal of participatory decision-making argued for by Irwin, Wynne and others, through facilitating participatory knowledge production. At the same time, following the ideas of Bonney and his colleagues, OPAL activities also deliver against more explicitly formulated science and education goals.

In the broadest sense, although conceived ahead of the recent upsurge of interest in the classification of citizen science, OPAL does closely follow the key design steps identified by Dickinson and Bonney [40] who proposed the following topics for consideration: choosing a scientific question; forming a project team; developing and refining project materials; recruiting and training participants; accepting, editing and displaying data; analysing and interpreting data, disseminating results, measuring impacts. Whereas in OPAL to date, the research questions and analysis of results have been almost exclusively the province of professional scientists, the national survey series was explicitly designed to engage the widest possible audience in data gathering.

Despite the manifold faces of citizen science, the ever evolving discipline unites academics, educators, community members and policy makers and delivers a raft of benefits for both research and outreach. As we have seen, the approach taken when establishing, designing and delivering citizen science projects can be diverse and deliver a host of different outcomes.This field is evolving rapidly, driven by new technology and experience gained from professional scientists and the public alike as they participate in and contribute to our understanding of CS through projects such as OPAL.

## Abbreviations

apps: applications
APRIL: Air Pollution Research in London
CS: citizen science
FSC: Field Studies Council
GPS: Global Positioning System
ICL: Imperial College London
NBN: National Biodiversity Network
NHM: Natural History Museum
OPAL: Open Air Laboratories
OU: Open University
